# Anxiety and Sleep Disturbances Among Health Care Workers During the COVID-19 Pandemic in India: Cross-Sectional Online Survey

**DOI:** 10.2196/24206

**Published:** 2020-12-22

**Authors:** Bhawna Gupta, Vyom Sharma, Narinder Kumar, Akanksha Mahajan

**Affiliations:** 1 Torrens University Public Health Melbourne Australia; 2 Military Hospital and Spinal Cord Injury Centre Pune India; 3 Medanta Hospital Lucknow India; 4 Monash University Melbourne Australia

**Keywords:** occupational epidemiology, anxiety, GAD-7, sleep quality, health care worker, pandemic, COVID-19, online survey, sleep, mental health, personal protective equipment

## Abstract

**Background:**

The COVID-19 pandemic caused by SARS-CoV-2 has become a serious concern among the global medical community and has resulted in an unprecedented psychological impact on health care workers, who were already working under stressful conditions.

**Objective:**

In this study, we aimed to evaluate and measure the effects of the COVID-19 pandemic on the anxiety levels and sleep quality among health care workers in India, as well as to determine how the unavailability of personal protective equipment affects their willingness to provide patient-related care.

**Methods:**

We conducted an online cross-sectional study using piloted, structured questionnaires with self-reported responses from 368 volunteer male and female health care workers in India. Study participants were identified through social networking platforms such as Facebook and WhatsApp. The survey evaluated the participants’ degree of signs and symptoms of anxiety and sleep quality based on the 7-item Generalized Anxiety Disorder (GAD-7) scale and single-item Sleep Quality Scale, respectively. Information on the availability of personal protective equipment was collected based on responses to relevant survey questions.

**Results:**

The majority of health care workers (126/368, 34.2%) were in the age group 45-60 years, and 52.2% (192/368) were doctors. Severe anxiety (ie, GAD-7 score >10) was observed among 7.3% (27/368) health care workers, whereas moderate, mild, and minimal anxiety was observed among 12.5% (46/368), 29.3% (108/368), and 50.8% (187/368) health care workers, respectively. Moreover, 31.5% (116/368) of the health care workers had poor-to-fair sleep quality (ie, scores <6). Univariate analysis showed female gender and inadequate availability of personal protective equipment was significantly associated with higher anxiety levels (*P*=.01 for both). Sleep disturbance was significantly associated with age <30 years (*P*=.04) and inadequate personal protective equipment (*P*<.001). Multivariable analysis showed that poorer quality of sleep was associated with higher anxiety levels (*P*<.001).

**Conclusions:**

The COVID-19 pandemic has potentially caused significant levels of anxiety and sleep disturbances among health care workers, particularly associated with the female gender, younger age group, and inadequate availability of personal protective equipment. These factors put health care workers at constant risk of contracting the infection themselves or transmitting it to their families. Early identification of at-risk health care workers and implementation of situation-tailored mitigation measures could help alleviate the risk of long-term, serious psychological sequelae as well as reduce current anxiety levels among health care workers.

## Introduction

An outbreak of pneumonia of unknown origin occurred in Hubei Province in China in December 2019, and owing to its ease of transmission, it has raised several concerns across more than 200 countries, areas, and territories worldwide. The global spread of COVID-19 has resulted in the World Health Organization (WHO) declaring COVID-19 as a pandemic [1,2]. Unprecedented international and national government strategies, including a strict lockdown in India, resulted in a slow albeit steady spread of COVID-19 in India as well as other countries. However, the phased relaxation of lockdown and resumption of economic activities have now led to an explosive surge in the number of COVID-19 cases in India, with a tally of 7,494,551 cases and 114,031 resulting deaths as of October 19, 2020 [2].

Previous studies provide evidence that COVID-19 has severe impact on psychological stressors, fear and anxiety, and poor sleep outcomes [3-6]. High anxiety levels during the pandemic have been strongly associated with functional impairments, alcohol or drug coping, negative religious coping, extreme hopelessness, and passive suicidal ideation [7]. Similarly, problematic sleep is associated with adverse consequences on the patient’s psychological, social, and cognitive functioning, which leads to deterioration of the overall quality of life [8].

Health care workers (HCWs, including doctors, nurses, dentists, and paramedics) are regarded as the saviors of human life; nevertheless, they remain wounded by the psychological consequences of COVID-19. Frontline workers in particular, who are directly involved in management of patients with COVID-19, are at a greater risk than others [9-11]. Initial estimates suggest that frontline HCWs account for 10%-20% of all COVID-19 diagnoses [12].

In India, with a population of approximately 1.3 billion [13] and a doctor-population ratio of 1:1800 [14], the already inadequate public health care system has crumbled during the COVID-19 pandemic, further pushing frontline HCWs to the edge. Moreover, HCWs are vulnerable to physical and psychological fatigue and poor sleep outcomes due to increased workload, physical exhaustion with irregular work schedules, frequent work shifts [15], and the occasional need to make ethically challenging decisions, including rationing of care [16-18].

They are also constantly challenged by isolation and live with an omnipresent fear of contracting the infection themselves or transmitting it to their families. This fear seems to be a major factor causing a psychological impact among HCWs, apart from separation from families, shortage of personal protective equipment (PPE), lack of essential intensive care units, as well as universally and rapidly changing guidelines on disease transmission and treatment that further add to their stress. Multiple cognitive behavioral theoretical models have suggested that the following factors contribute to the severity of health anxiety: memory and attention process, misinterpretation of health-related stimuli, and maladaptive beliefs and behaviors [19].

Research on HCWs has revealed that approximately 50% of physicians have reported poor sleep quality during the pandemic, which may be attributed to the contagious nature of COVID-19 [15,20] and the emergency nature of their work [15]. Subjective sleep quality is defined by the satisfaction of one’s overall sleep experience, including aspects of sleep initiation, sleep maintenance, sleep quantity, and refreshment upon awakening [18].

The Indian perspective on anxiety, sleep outcomes, and the availability of PPE among HCWs fighting the COVID-19 pandemic is sparse. Previous studies have provided some evidence, including a study in India that evaluated stress levels only among orthopedic surgeons by using a self-validated scale and another study that assessed anxiety and depression only among doctors and nurses [21,22]. The Indian health infrastructure is not robust enough to address and provide coping strategies for its HCWs. Moreover, significant demand and supply chain disruptions have prompted efforts to conserve the limited PPE available through extended use or reuse, thereby adding an additional source of risk and anxiety for HCWs [23].

Therefore, the true burden of psychological impact needs to be measured, and it is paramount for all levels of health care organizations to screen for HCWs who are at risk of being affected by unprecedented events such as the COVID-19 pandemic. To our knowledge, this is the first study to assess the burden of anxiety disorders and effect on sleep quality of Indian HCWs during the COVID-19 pandemic. We also evaluated the extent to which the unavailability of PPE affects the readiness of HCWs to perform patient-related care.

## Methods

### Study Design

We performed a cross-sectional online study with HCWs. An online survey was preferred to the traditional method of data collection, as it increases the willingness of participants to answer anxiety-driven or sensitive questions [24] and avoid standard, socially desirable responses [25]. Additionally, it allows for cost-effective and instantaneous data transmission and flexibility in participation at any time of the day or night, in turn, allowing for increased response rates.

### Study Participants

The inclusion criteria for this study were (1) male and female HCWs; (2) full-time practicing doctors, nurses, dentists, and paramedic staff directly involved in providing any kind of patient-related care, including but not limited to triage, screening, diagnosis, and treatment; (3) aged over 18 years and residents of India; (4) employed at either public or private hospitals or clinics; and (5) well-versed in the English language. The exclusion criteria were HCWs who were not on active medical duty at the time of the survey due to any leave of absence.

### Sample and Data Collection

Facebook and WhatsApp were used as the sampling frame. We used a respondent-driven probability sampling method, which draws on Facebook’s inherent peer network structures to encourage users to recruit other HCWs who may or may not be on Facebook [26]. The diversity of Facebook members lends itself to a stratified sampling approach that may increase the representativeness of the sample. Study participants were invited through 3 online venues: (1) public or “wall” posts on Facebook advertising our research survey on popular HCW groups (ie, virtual communities linking people with some shared interest, attribute, or cause), (2) peer referral on Facebook, and (3) WhatsApp messages sent from May 2 to May 16, 2020.

Facebook advertisement for our online survey included a headline describing the survey, a brief scholarly review of the literature on the impact of COVID-19 on HCWs, and contact details of the principal investigator. The advertisement also contained an electronic link to the research questionnaire and mentioned the time required to complete the survey (ie, approximately 5 minutes). A hyperlink to the survey was also shared.

The advertisement to participate in the survey was reposted every 3 days across a period of 2 weeks. This strategy resulted in the advertisement reappearing on the users’ “news feed.” It also allowed the advertisement to be posted on our friends’ walls, as well as in the feeds of their friends, including those who may not be friends with us on Facebook. We also sent private messages via Facebook and WhatsApp, to remind HCWs to participate in the survey. Furthermore, we created a Facebook page to promote our research and sent out mass messages addressing the HCWs among the members of this Facebook page.

### Ethical Approval

This study was performed in accordance with the ethical standards laid down in the 1964 Declaration of Helsinki and its later amendments. Ethical approvals were obtained from the ethics committee of the Armed Forces Medical College, Pune (IEC/2020/154).

We included a disclaimer in our Facebook advertisement that participation in the survey was completely voluntary and survey responses were anonymous; thus, confidentiality of all participants was fully respected. Furthermore, electronic informed consent was obtained from each participant before the start of the survey. Participants had the right to withdraw from the study at any stage without any justification. They were not provided with any incentives. Moreover, participants had the opportunity to contact the investigators of this study via Facebook or WhatsApp for any questions or concerns.

### Survey Questionnaire

The self-reported online survey was created using Google Forms and included 4 sections as described below. We conducted a pilot test of the questionnaire on the first 20 study participants, and their data have not been used in this study. Please refer Multimedia Appendix 1 for the questionnaire.

#### Generalized Anxiety Disorder

The 7-item Generalized Anxiety Disorder (GAD-7) scale is a valid and efficient tool for screening anxiety and assessing its severity in clinical practice. It consists of 7 multiple-choice questions [27,28] that assess the frequency of anxiety symptoms over the past 2 weeks on a 4-point Likert scale, with scores of 0, 1, 2, and 3 assigned to the response categories “not at all,” “several days,” “more than half the days,” and “nearly every day,” respectively. The total GAD-7 score for the 7 items ranges from 0 to 21, with increasing scores indicating more severe functional impairments as a result of anxiety. In this study, we considered GAD-7 scores of 5, 10, and 15 as the cut-off points for mild, moderate, and severe anxiety, respectively. With a threshold score of 10, a previous study reported 89% sensitivity and 82% specificity for GAD-7 [27].

#### Sleep Quality Scale

A validated simple, practical, and pragmatic single-item sleep quality scale (SQS) was used to assess sleep quality [29]. It helps measure sleep quality over a 7-day recall period whereby the study participants mark an integer score from 0 to 10, according to the following 5 categories: 0, terrible; 1-3, poor; 4-6, fair; 7-9, good; and 10, excellent. When using the SQS, participants were instructed to consider the following core components of sleep quality: how many hours of sleep they had, how easily they fell asleep, how often they woke up during the night (except to go to the bathroom), how often they woke up earlier than they had to in the morning, and how refreshing their sleep was.

#### Availability of PPE

Two of the survey questions were specifically designed to assess the participant’s anxiety about the lack of access to PPE while performing patient-related care. These questions were validated using the face validation and content validation techniques of Lawshe criterion (content validity ratio = 1) [30].

#### Demographic Information

Finally, participants’ demographic information, including age, gender, marital status, educational level, and type of health care profession was collected via some specific survey questions.

### Statistical Analysis

The descriptive analysis for demographic data and preparedness for COVID-19–related questions were expressed in percentages. The relationship between 2 categorical variables, that is, anxiety among doctors and nurses regarding age and availability of PPE were estimated using chi-square test. Multivariable analysis was used to correlate the factors influencing the prevalence of anxiety of varying severity and sleep quality, with participants’ age group, gender, educational level, marital status, profession, and place of work, and availability of PPE. *P* value, odds ratio (OR), and 95% CI were determined to indicate statistical significance. Hosmer–Lemeshow index was used to assess the overall model fit. A 2-tailed *P* value of <.05 indicated statistical significance. Data were analyzed using SPSS software (v.20; IBM Corp).

## Results

### Demographic Characteristics

Table 1 shows the demographic characteristics of the survey participants (N=368). In all, 46% (168/368) were male and 54.3% (200/368) were female HCWs. Majority of the participants (227/368, 61.7%) were working in a tertiary care facility. A higher proportion of HCWs reported the lack of availability of satisfactory PPE (140/368, 38%) than those who reported availability of PPE (129/368, 35.1%).

**Table 1:** Demographic characteristics of the study population (N=368).

Characteristics		Participants, n (%)
**Gender**		
	Male	168 (45.7)
	Female	200 (54.3)
**Age (years)**		
	<30	89 (24.2)
	30-44	108 (29.3)
	45-60	126 (34.2)
	>60	45 (12.2)
**Marital status**		
	Unmarried	82 (22.3)
	Married	139 (37.8)
	Married with children	145 (39.4)
	Divorced	2 (0.5)
**Education level**		
	High school	15 (4.1)
	Graduate	161 (43.8)
	Postgraduate	160 (43.4)
	Doctorate	32 (8.6)
**Profession**		
	Doctor	192 (52.2)
	Nurse	140 (38)
	Dentist	24 (6.5)
	Paramedic	12 (3.2)

### Comparative Analyses

Table 2 presents the comparative analysis of anxiety among doctors and nurses with regard to age (years) and availability of PPE. HCWs with moderate (GAD-7 score = 10) and severe (GAD-7 score = 15) anxiety scores were considered as “high risk” for anxiety for the purpose of this analysis. The results were not statistically significant for any age group and the lack of availability of adequate-quality PPE with respect to the anxiety levels among both doctors and nurses.

**Table 2:** Anxiety levels among doctors and nurses with regard to age and availability of personal protective equipment, determined based on Generalized Anxiety Disorder, 7-item (GAD-7) score.

Variable		Anxiety among doctors				Anxiety among nurses		
		High risk^a^	Low risk^b^	*P* value	High risk		Low risk	*P* value
**Age (years), n (%)**				.75				.07
	<30	8 (24.2)	25 (75.7)		7 (15.2)		39 (84.7)	
	30-44	10 (17.2)	48 (82.7)		11 (25.5)		32 (74.4)	
	45-60	12 (15.7)	64 (84.2)		2 (5.5)		34 (94.4)	
	>60	5 (20)	20 (80)		1 (6.6)		14 (93.3)	
**Availability of adequate-quality PPE^c^**				.13				.16
	PPE not required	1 (7.6)	12 (92.3)		1 (9.09)		10 (90.9)	
	Yes	11 (14.8)	63 (85.1)		3 (6.3)		43 (93.6)	
	No	19 (26.3)	53 (73.6)		11 (20.3)		44 (79.6)	
	Not sure	4 (12.1)	29 (87.8)		6 (21.4)		22 (78.5)	

^a^GAD-7 score >10.

^b^GAD-7 score <10.

^c^PPE: personal protective equipment.

### Univariate Analysis

Table 3 describes factors affecting anxiety and sleep quality among HCWs. Half of the HCWs (187/368, 50.8%) had minimal anxiety as determined by the GAD-7 scores. Mild, moderate, and severe anxiety levels were observed among 29.3% (108/368), 12.5% (46/368), and 7.3% (27/368) of HCWs, respectively. The sleep score was poor for 5.7% (21/368) and fair for 25.8% (95/368) HCWs, reflecting significant sleep disturbance. In contrast, good and excellent sleep scores were observed for 56.3% (207/368) and 12.2% (45/368) HCWs.

Male HCWs had significantly minimal anxiety scores (219/368, 59.5%) than female HCWs. Moreover, there was a significant association between the female gender as well as inadequate availability of PPE and higher anxiety levels (*P*=.01 for both).

**Table 3:** Factors affecting anxiety and quality of sleep among health care workers (N=368).

Variables		Total	Anxiety level (based on GAD-7^a^ score)					Quality of sleep					
			Minimal (0-4)	Mild (5-9)	Moderate (10-14)	Severe (15-21)	*P* value		Poor (0-3)	Fair (4-6)	Good (7-9)	Excellent (10)	*P* value
**Age (years), n (%)**							.19						<.001
	<30	89 (24.2)	44 (49.4)	26 (29.2)	13 (14.6)	6 (6.7)			6 (6.7)	33 (37.1)	43 (48.3)	7 (7.9)	
	30-44	108 (29.3)	43 (39.8)	38 (35.2)	15 (13.9)	12 (11.1)			9 (8.3)	22 (20.4)	68 (63)	9 (8.3)	
	45-60	126 (34.2)	71 (56.3)	34 (27)	15 (11.9)	6 (4.8)			3 (2.4)	35 (27.8)	70 (55.6)	18 (14.3)	
	>60	45 (12.2)	29 (64.4)	10 (22.2)	3 (6.7)	3 (6.7)			3 (6.7)	5 (11.1)	26 (57.8)	11 (24.4)	
**Gender**							.01						.54
	Male	168 (45.7)	100 (59.5)	37 (22)	20 (11.9)	11 (6.5)			9 (5.4)	41 (24.4)	93 (55.4)	25 (14.9)	
	Female	200 (54.3)	87 (43.5)	71 (35.5)	26 (13)	16 (8)			12 (6)	54 (27)	114 (57)	20 (10)	
**Marital Status**							.41						.22
	Unmarried	82 (22.2)	40 (48.8)	25 (30.5)	10 (12.2)	7 (8.5)			4 (4.9)	30 (36.6)	40 (48.8)	8 (9.8)	
	Married	139 (37.7)	80 (57.6)	34 (24.5)	17 (12.2)	8 (5.8)			11 (7.9)	31 (22.3)	83 (59.7)	14 (10.1)	
	Married with Children	145 (39.4)	67 (46.2)	47 (32.4)	19 (13.1)	12 (8.3)			6 (4.1)	34 (23.4)	82 (56.6)	23 (15.9)	
	Divorced	2 (0.5)	0	2 (100)	0	0			0	0	2 (100)	0	
**Education level**							.34						.35
	High school	15 (4.1)	6 (40)	7 (46.6)	2 (13.3)	0			1 (6.6)	5 (33.3)	8 (53.3)	1 (6.6)	
	Graduate	161 (43.7)	81 (50.3)	51 (31.6)	19 (11.8)	10 (6.2)			9 (5.5)	51 (31.6)	80 (49.6)	21 (13)	
	Postgraduate	160 (43.4)	78 (48.7)	44 (27.5)	23 (14.3)	15 (9.3)			11 (6.8)	32 (20)	98 (61.2)	19 (11.8)	
	Doctorate	32 (8.6)	22 (68.75)	6 (18.7)	2 (6.2)	2 (6.2)			0	7 (21.8)	21 (65.6)	4 (12.5)	
**Profession**							.14						.44
	Doctor	192 (52.2)	109 (56.8)	46 (24)	23 (12)	14 (7.3)			14 (7.3)	43 (22.4)	109 (56.8)	26 (13.5)	
	Dentist	24 (6.5)	9 (37.5)	6 (25)	6 (25)	3 (12.5)			1 (4.2)	10 (41.7)	11 (45.8)	2 (8.3)	
	Nurse	140 (38)	62 (44.3)	53 (37.9)	16 (11.4)	9 (6.4)			6 (4.3)	39 (27.9)	81 (57.9)	14 (10)	
	Paramedics	12 (3.3)	7 (58.3)	3 (25)	1 (8.3)	1 (8.3)			0	3 (25)	6 (50)	3 (25)	
**Place of work**							.34						.64
	Primary	59 (16)	26 (44.1)	18 (30.5)	9 (15.3)	6 (10.2)			4 (6.8)	12 (20.3)	34 (57.6)	9 (15.3)	
	Secondary	68 (18.4)	40 (58.8)	17 (25)	4 (5.9)	7 (10.3)			4 (5.9)	20 (29.4)	40 (58.8)	4 (5.9)	
	Tertiary	229 (62.2)	114 (49.8)	71 (31)	30 (13.1)	14 (6.1)			13 (5.7)	59 (25.8)	128 (55.9)	29 (12.7)	
	Not a health care facility	12 (3.2)	7 (58.3)	2 (16.7)	3 (25)	0			0	4 (33.3)	5 (41.7)	3 (25)	
**Availability of adequate-quality PPE^**b**^**							.01						<.001
	Yes	131 (35.5)	83 (63.4)	30 (22.9)	12 (9.2)	6 (4.6)			5 (3.8)	28 (21.4)	81 (61.8)	17 (13)	
	No	140 (38)	58 (41.4)	42 (30)	24 (17.1)	16 (11.4)			13 (9.3)	39 (27.9)	78 (55.7)	10 (7.1)	
	Not Sure	66 (17.9)	28 (42.4)	26 (39.4)	7 (10.6)	5 (7.6)			3 (4.5)	21 (31.8)	35 (53)	7 (10.6)	
	PPE not required	31 (8.4)	18 (58.1)	10 (32.3)	3 (9.7)	0			0	7 (22.6)	13 (41.9)	11 (35.5)	

^a^GAD-7: Generalized Anxiety Disorder, 7-item (GAD-7) scale.

^b^PPE: personal protective equipment.

### Multivariable Regression Analysis

Table 4 shows a negative association between gender, marital status, education, and availability of PPE; however, these were not statistically significant (*P*=.21, .83, .05, and .09 respectively). Similarly, there was no significant association between age and place of work as a factor for higher anxiety levels. A similar pattern of association was observed for sleep disturbance and the abovementioned factors, with no statistically significant association for poor sleep scores (Table 4).

Furthermore, the correlation of anxiety scores and quality of sleep scores showed a significant inverse relation, reflecting poorer quality of sleep as the GAD-7 score increased (*P*<.001; Table 5).

**Table 4:** Factors influencing anxiety and sleep quality among health care workers.

Variables			Anxiety symptoms (GAD-7^a^ score >10)								Sleep quality score^b^ ≤3						
			β		SE		Adjusted odds ratio (95% CI)		*P* value		β		SE		Adjusted odds ratio (95% CI)		*P* value
**Age (years)**									.36								.20
	>45	ref^c^		ref		1				ref		ref		1			
	<45	.31		0.35		1.37 (0.69-2.71)				.79		0.62		2.214 (0.66-7.43)			
**Gender**									.21								.72
	Female	ref		ref		1				−.18		0.50		1			
	Male	−.39		0.31		0.68 (0.37-1.25)				ref		ref		0.84 (0.31-2.25)			
**Marital status**									.84								.95
	Other	ref		ref		1				ref		ref		1			
	Married or married with children	−.04		0.18		0.96 (0.67-1.38)				.02		0.30		0.98 (0.54-1.76)			
**Education**									.06								.56
	Lower than graduate	ref		ref		1				ref		ref		1			
	Graduate and above	−.56		0.30		0.57 **(**0.32-1.02)				−.26		0.47		0.77 (0.30-1.93)			
**Place of work**									.30								.45
	Not primary HCW^d^	ref		ref		1				ref		ref		1			
	Primary HCW	.18		0.17		1.19 (0.85-1.67)				.21		0.28		1.23 (0.72-2.11)			
**PPE^e^ availability**									.09								.78
	No	ref		ref		1^f^				−.18		0.63		1			
	Yes	−.63		0.37		0.53 (0.25-1.10)				ref		ref		0.84 (0.24-2.86)			

^a^GAD-7 score ≥10 indicates moderate-to-severe anxiety symptoms.

^b^Sleep quality score ≤3 indicates terrible-to-poor sleep quality.

^c^ref: reference value.

^d^HCW: health care worker.

^e^Personal protective equipment.

^f^1 indicates the reference value.

**Table 5:** Correlation of Generalized Anxiety Disorder, 7-item (GAD-7) scores and quality of sleep scores among health care workers.

GAD-7^a^ score	Sleep quality scale^b^				Total	
	Poor (1-3)	Fair (4-6)	Good (7-9)	Excellent (10)		
Minimal (0-4)	9	25	117	36		187
Mild (6-9)	4	39	60	5		108
Moderate (10-14)	3	16	25	2		46
Severe (15-21)	5	15	5	2		27
Total	21	95	207	45		368

^a^GAD-7: Generalized Anxiety Disorder, 7-item scale.

^b^The correlation is significant at *P*<.001.

## Discussion

### Principal Findings

This is the first online survey conducted on HCWs in India during the COVID-19 pandemic that highlights the potential burden of anxiety, sleep outcomes, and the impact of inadequate availability of PPE. Stress and symptoms of anxiety among HCWs is not a new phenomenon, as there is ample literature on burnout among HCWs during routine professional work [31,32]. This study’s findings are consistent with previous SARS outbreaks wherein HCWs reported high levels of fear of contagion and transmitting infection to their family members, in addition to emotional disturbance, uncertainty, and stigmatization [33-37]. This paradigm of anxiety among HCWs when analyzed from the perspective of a global emergency such as the COVID-19 pandemic requires a unique approach each time.

The results of our study reflect similar trends observed in surveys conducted in China [17,38]. However, these previous studies reported relatively fewer HCWs with severe anxiety levels. This may be due to the temporal association of the timing of the surveys since these were conducted in January and February 2020, which was before the WHO’s declaration of COVID-19 as a pandemic and a global health emergency. Pappa et al [39] also reported noteworthy features of anxiety and poor sleep outcomes among HCWs in their meta-analysis.

Compared to male participants, female participants found it more challenging to manage their anxiety levels, particularly regarding the inadequate availability of PPE during the COVID-19 pandemic. These results are corroborated by a UK study conducted on orthopedic members [40]. The underlying association of female gender with a higher incidence of generalized anxiety across different countries was evident in a systematic review by Remes et al [41]. The higher anxiety scores among female participants in our study could be attributed to factors such as insecurity at the workplace and mental pressure to fulfil domestic responsibilities. The differentiation of anxiety scores among doctors and nurses based on gender was not significant, which reiterates the association of the female gender with higher anxiety in situations such as the COVID-19 pandemic. Cai et al [42] comprehensively evaluated the psychological health of frontline HCWs working in Hunan province and found that female HCWs were more likely to develop effective social and personal coping strategies to mitigate stress.

In addition to the innate factors responsible for higher anxiety among females, another stressor for HCWs is the “well-being” of their families [10]. This was reflected in our study as higher anxiety scores observed among HCWs who were either married or married with children than those among unmarried HCWs, although the difference was not statistically significant. An online survey conducted by University of Arkansas for Medical Sciences to assess and ensure “well-being” of their physicians found that the primary worry of all HCWs was the safety of their families during the COVID-19 pandemic, which was regarded as a major anxiety stress factor [43]. These findings highlight the need for future studies with larger sample sizes to further explore this association.

The lack of adequate PPE, which poses a threat to self as well as the family of HCWs, was another significant factor associated with higher levels of anxiety and poor sleep outcomes. A significant number of HCWs, with the majority of them being female, admitted to the unavailability of adequate PPE resulting in a highly significant correlation with poor sleep scores and moderate-to-severe anxiety levels on the GAD-7 scale. Similar results about poor sleep outcomes among female participants have been reported in other studies [6,44]. The negative psychological impact of inadequate as well as poor-quality PPE has been previously analyzed in the context of the health care systems in the United States of America and Asia [42,43,45]. The pivotal role of the health care administrative machinery in assuring timely provision of adequate and good-quality PPE is an essential mitigation strategy that can lower anxiety levels among HCWs. This has been implemented in Singapore and applauded globally [46].

Within the context of the Indian health care system, HCWs’ education and work facility do not seem to be significant stressors even during this pandemic. A possible explanation for this observation could be their past experience of working in an already stretched and an overwhelmed health care system, which may have initiated a chain of events that might lead to them coping better in such extreme scenarios. However, we observed a trend of higher anxiety scores and poor sleep scores for HCWs employed at the primary care level that can emanate from a lack of security at workplace or unsafe working conditions and, indirectly, unavailability of adequate PPE. In addition to the enormous patient burden, an intense lockdown imposed by the government could be a trigger for anxiety for HCWs due to apprehension of closure of smaller private clinics and the issue of sustainability as compared to a tertiary or corporate health care setting.

HCWs who manifest signs and symptoms of anxiety have been observed with signs of insomnia, which is reflected by their poor or fair sleep scores. Similar findings were reported in a web-based study conducted in China [3]. Pappa et al [39], in their systematic review, emphasized the clustering of such symptoms that has been evident during the COVID-19 pandemic. Our study also revealed a similar pattern of statistically significant correlation between moderate-to-severe anxiety on the GAD-7 scale and poor-to-fair sleep scores. The conversion of one symptom to another remains a matter of debate that has been observed in earlier major outbreaks like SARS as well [47,48], although COVID-19 has surpassed the SARS outbreak with regard to case load as well as global mortality.

### Future Recommendations

The adverse effects of this and many other studies highlight the need for additional multipronged psychological support [10] to be provided by hospitals and organizations employing HCWs. The stress-adaptation model may be useful when considering suitable interventions. This model describes the experience of stress as a universal response to extraordinary events and environments. Under this model, a range of normal reactions such as anxiety, depression, and sleep disturbance should not be considered pathological, but instead, reframed and realigned as adaptation mechanisms. Past strategies employed during the SARS outbreak include confidence building, distribution of informational pamphlets describing signs and support resources for anxiety and stress, as well as availability of “drop in” sessions and confidential telephone support with psychiatric staff [34]. Adequate care, emotional support, and motivation should be given to HCWs by their family and community members, as this has shown to have a positive impact on sleep outcomes and may promote adaptive coping strategies [49,50]. Moreover, immediate supervisors should always be open for communication with an empathetic attitude towards HCWs [51].

In addition, there should be better community awareness to reduce social stigma regarding mental health [52]. Despite an extremely fragile and overburdened health care system in India, the government has initiated certain exemplary confidence-building initiatives such as an insurance scheme worth an approximate amount of US $ 66,670 for HCWs fighting in the COVID-19 crisis [53].

### Strengths and Limitations

A major strength of our study is that our survey was based on previously validated and well-established objective tools such as GAD-7 and SQS for the assessment of anxiety and sleep outcomes, respectively [28]. The online modality of our cross-sectional survey prevented the risk of COVID-19 infection via droplet or contact transmission. Furthermore, the use of respondent-driven sampling has greater external validity, as it extends the sample to all potential members of the subgroups by reaching out to participants through their social networks. This sampling method also allows reaching out to some HCWs who may otherwise be undiscoverable and challenging to reach by phone or face-to-face interviews [54].

There was a proportionate representation of nurses as well as doctors among the respondent HCWs in this study; this would likely mitigate the bias of having a higher number of doctors as in previous studies conducted in India [21]. Moreover, this study highlights the prevalence of anxiety signs and symptoms as well as poor sleep quality in a wide spectrum of HCWs during this crucial period.

Nevertheless, this study has some limitations, including the small size convenience sample collected over a 2-week period, which limits the generalizability of the results to all HCWs across India. We also acknowledge the selection and self-reporting bias in this study, which depends on the cognitive capabilities of the respondent, the disposition of the respondent towards socially desirable responses, and situational and task-related conditions [55].

### Conclusions

This study highlights the psychological impact of the COVID-19 pandemic on frontline workers. The pandemic has led to features of generalized anxiety and poor sleep quality that is significantly associated with factors such as the female gender and availability of PPE. These findings underscore the need to identify HCWs at risk at an early stage and enable comprehensive, tiered, as well as situation-tailored mitigation measures, enhancing the HCWs’ psychological resilience and alleviating their vulnerability in the present pandemic conditions. Limitations of working hours, special training to manage patients with COVID-19, availability of adequate-quality PPE, along with timely and appropriate mental health support through multidisciplinary teams are vital elements of such mitigation measures. These efforts are essential because of the impact of anxiety on not only HCWs’ personal well-being but also on health care delivery overall, which may be affected by the HCWs’ potentially impaired decision-making ability, judgement, and attention.

## Appendix

Multimedia Appendix 1 Questionnaire administered to health care workers in this study.

## Abbreviations

GAD-7: Generalized Anxiety Disorder 7-item (scale)
HCW: health care worker
PPE: personal protective equipment
SQS: sleep quality scale
WHO: World Health Organization

## References

[1] Cucinotta D, Vanelli M (2020). WHO declares COVID-19 a pandemic. Acta Biomed.

[2] WHO Coronavirus Disease (COVID-19) Dashboard. World Health Organization.

[3] Huang Y, Zhao N (2020). Generalized anxiety disorder, depressive symptoms and sleep quality during COVID-19 outbreak in China: a web-based cross-sectional survey. Psychiatry Res.

[4] Choi EPH, Hui BPH, Wan EYF (2020). Depression and anxiety in Hong Kong during COVID-19. Int J Environ Res Public Health.

[5] Ahorsu DK, Lin C, Imani V, Saffari M, Griffiths MD, Pakpour AH (2020). The fear of COVID-19 scale: development and initial validation. Int J Ment Health Addict.

[6] Jahrami H, BaHammam AS, AlGahtani H, Ebrahim A, Faris M, AlEid K, Saif Z, Haji E, Dhahi A, Marzooq H, Hubail S, Hasan Z (2020). The examination of sleep quality for frontline healthcare workers during the outbreak of COVID-19. Sleep Breath.

[7] Lee SA (2020). Coronavirus Anxiety Scale: A brief mental health screener for COVID-19 related anxiety. Death Stud.

[8] Szentkirályi András, Madarász Csilla Z, Novák Márta (2009). Sleep disorders: impact on daytime functioning and quality of life. Expert Rev Pharmacoecon Outcomes Res.

[9] Spoorthy MS, Pratapa SK, Mahant S (2020). Mental health problems faced by healthcare workers due to the COVID-19 pandemic-A review. Asian J Psychiatr.

[10] Shanafelt T, Ripp J, Trockel M (2020). Understanding and addressing sources of anxiety among health care professionals during the COVID-19 pandemic. JAMA.

[11] Esakandari H, Nabi-Afjadi M, Fakkari-Afjadi J, Farahmandian N, Miresmaeili S, Bahreini E (2020). A comprehensive review of COVID-19 characteristics. Biol Proced Online.

[12] Nguyen LH, Drew DA, Graham MS, Joshi AD, Guo C, Ma W, Mehta RS, Warner ET, Sikavi DR, Lo C, Kwon S, Song M, Mucci LA, Stampfer MJ, Willett WC, Eliassen AH, Hart JE, Chavarro JE, Rich-Edwards JW, Davies R, Capdevila J, Lee KA, Lochlainn MN, Varsavsky T, Sudre CH, Cardoso MJ, Wolf J, Spector TD, Ourselin S, Steves CJ, Chan AT, COronavirus Pandemic Epidemiology Consortium (2020). Risk of COVID-19 among front-line health-care workers and the general community: a prospective cohort study. Lancet Public Health.

[13] India Population (LIVE). Worldometer.

[14] Deo MG (2013). "Doctor population ratio for India - the reality". Indian J Med Res.

[15] Zhang C, Yang L, Liu S, Ma S, Wang Y, Cai Z, Du H, Li R, Kang L, Su M, Zhang J, Liu Z, Zhang B (2020). Survey of insomnia and related social psychological factors among medical staff involved in the 2019 novel coronavirus disease outbreak. Front Psychiatry.

[16] Chan-Yeung M (2004). Severe acute respiratory syndrome (SARS) and healthcare workers. Int J Occup Environ Health.

[17] Lai J, Ma S, Wang Y, Cai Z, Hu J, Wei N, Wu J, Du H, Chen T, Li R, Tan H, Kang L, Yao L, Huang M, Wang H, Wang G, Liu Z, Hu S (2020). Factors associated with mental health outcomes among health care workers exposed to coronavirus disease 2019. JAMA Netw Open.

[18] Kline C, Gellman MD, Turner JR (2013). Encyclopedia of Behavioral Medicine.

[19] Taylor S (2019). The Psychology of Pandemics: Preparing for the next global outbreak of infectious disease.

[20] Qiu D, Yu Y, Li R, Li Y, Xiao S (2020). Prevalence of sleep disturbances in Chinese healthcare professionals: a systematic review and meta-analysis. Sleep Med.

[21] Wilson W, Raj JP, Rao S, Ghiya M, Nedungalaparambil NM, Mundra H, Mathew R (2020). Prevalence and predictors of stress, anxiety, and depression among healthcare workers managing COVID-19 pandemic in India: a nationwide observational study. Indian J Psychol Med.

[22] Sahu D, Agrawal T, Rathod V, Bagaria V (2020). Impact of COVID 19 lockdown on orthopaedic surgeons in India: A survey. J Clin Orthop Trauma.

[23] Livingston E, Desai A, Berkwits M (2020). Sourcing personal protective equipment during the COVID-19 pandemic. JAMA.

[24] Tourangeau R (2004). Survey research and societal change. Annu Rev Psychol.

[25] Chang L, Krosnick J (2009). National surveys via RDD telephone interviewing versus the internet: comparing sample representativeness and response quality. Public Opin Q.

[26] Heckathorn DD (1997). Respondent-driven sampling: a new approach to the study of hidden populations. Social Problems.

[27] Spitzer RL, Kroenke K, Williams JBW, Löwe Bernd (2006). A brief measure for assessing generalized anxiety disorder: the GAD-7. Arch Intern Med.

[28] Löwe Bernd, Decker O, Müller Stefanie, Brähler Elmar, Schellberg D, Herzog W, Herzberg PY (2008). Validation and standardization of the Generalized Anxiety Disorder Screener (GAD-7) in the general population. Med Care.

[29] Snyder E, Cai B, DeMuro C, Morrison MF, Ball W (2018). A new single-item sleep quality scale: results of psychometric evaluation in patients with chronic primary insomnia and depression. J Clin Sleep Med.

[30] Ayre C, Scally AJ (2017). Critical values for Lawshe’s content validity ratio. Measurement and Evaluation in Counseling and Development.

[31] Rotenstein LS, Torre M, Ramos MA, Rosales RC, Guille C, Sen S, Mata DA (2018). Prevalence of burnout among physicians: a systematic review. JAMA.

[32] Dyrbye LN, Shanafelt TD, Johnson PO, Johnson LA, Satele D, West CP (2019). A cross-sectional study exploring the relationship between burnout, absenteeism, and job performance among American nurses. BMC Nurs.

[33] Maunder RG, Lancee WJ, Balderson KE, Bennett JP, Borgundvaag B, Evans S, Fernandes CMB, Goldbloom DS, Gupta M, Hunter JJ, McGillis Hall L, Nagle LM, Pain C, Peczeniuk SS, Raymond G, Read N, Rourke SB, Steinberg RJ, Stewart TE, VanDeVelde-Coke S, Veldhorst GG, Wasylenki DA (2006). Long-term psychological and occupational effects of providing hospital healthcare during SARS outbreak. Emerg Infect Dis.

[34] Maunder R, Hunter J, Vincent L, Bennett J, Peladeau N, Leszcz M, Sadavoy J, Verhaeghe LM, Steinberg R, Mazzulli T (2003). The immediate psychological and occupational impact of the 2003 SARS outbreak in a teaching hospital. CMAJ.

[35] Wu KK, Chan SK, Ma TM (2005). Posttraumatic stress after SARS. Emerg Infect Dis.

[36] Lee AM, Wong JGWS, McAlonan GM, Cheung V, Cheung C, Sham PC, Chu C, Wong P, Tsang KWT, Chua SE (2007). Stress and psychological distress among SARS survivors 1 year after the outbreak. Can J Psychiatry.

[37] Cao W, Fang Z, Hou G, Han M, Xu X, Dong J, Zheng J (2020). The psychological impact of the COVID-19 epidemic on college students in China. Psychiatry Res.

[38] Zhang W, Wang K, Yin L, Zhao W, Xue Q, Peng M, Min B, Tian Q, Leng H, Du J, Chang H, Yang Y, Li W, Shangguan F, Yan T, Dong H, Han Y, Wang Y, Cosci F, Wang H (2020). Mental health and psychosocial problems of medical health workers during the COVID-19 epidemic in China. Psychother Psychosom.

[39] Pappa S, Ntella V, Giannakas T, Giannakoulis VG, Papoutsi E, Katsaounou P (2020). Prevalence of depression, anxiety, and insomnia among healthcare workers during the COVID-19 pandemic: A systematic review and meta-analysis. Brain Behav Immun.

[40] Thakrar A, Raheem A, Chui K, Karam E, Wickramarachchi L, Chin K (2020). Trauma and orthopaedic team members’ mental health during the COVID-19 pandemic. Bone & Joint Open.

[41] Remes O, Brayne C, van der Linde Rianne, Lafortune L (2016). A systematic review of reviews on the prevalence of anxiety disorders in adult populations. Brain Behav.

[42] Cai H, Tu B, Ma J, Chen L, Fu L, Jiang Y, Zhuang Q (2020). Psychological impact and coping strategies of frontline medical staff in Hunan between January and march 2020 during the outbreak of coronavirus disease 2019 (COVID‑19) in Hubei, China. Med Sci Monit.

[43] Berg S (2020). Survey: Doctors’ big COVID-19 worry is keeping their families safe. AMA - Physician Health.

[44] ter Horst JP, de Kloet ER, Schächinger H, Oitzl MS (2012). Relevance of stress and female sex hormones for emotion and cognition. Cell Mol Neurobiol.

[45] Wang X, Zhang X, He J (2020). Challenges to the system of reserve medical supplies for public health emergencies: reflections on the outbreak of the severe acute respiratory syndrome coronavirus 2 (SARS-CoV-2) epidemic in China. Biosci Trends.

[46] Wong JEL, Leo YS, Tan CC (2020). COVID-19 in Singapore-current experience: critical global issues that require attention and action. JAMA.

[47] Koh D, Lim MK, Chia SE, Ko SM, Qian F, Ng V, Tan BH, Wong KS, Chew WM, Tang HK, Ng W, Muttakin Z, Emmanuel S, Fong NP, Koh G, Kwa CT, Tan KB, Fones C (2005). Risk perception and impact of Severe Acute Respiratory Syndrome (SARS) on work and personal lives of healthcare workers in Singapore: what can we learn?. Med Care.

[48] Lancee WJ, Maunder RG, Goldbloom DS, Coauthors for the Impact of SARS Study (2008). Prevalence of psychiatric disorders among Toronto hospital workers one to two years after the SARS outbreak. Psychiatr Serv.

[49] Kent de Grey RG, Uchino BN, Trettevik R, Cronan S, Hogan JN (2018). Social support and sleep: A meta-analysis. Health Psychol.

[50] Prati G, Pietrantoni L (2010). The relation of perceived and received social support to mental health among first responders: a meta-analytic review. J. Community Psychol.

[51] Greenberg N, Docherty M, Gnanapragasam S, Wessely S (2020). Managing mental health challenges faced by healthcare workers during covid-19 pandemic. BMJ.

[52] Mohindra R, Suri V, Bhalla A, Singh SM (2020). Issues relevant to mental health promotion in frontline health care providers managing quarantined/isolated COVID19 patients. Asian J Psychiatr.

[53] Pradhan Mantri Garib Kalyan Package: Insurance Scheme for Healthcare Workers Fighting COVID-19. Ministry of Health and Family Welfare.

[54] Brickman Bhutta C (2012). Not by the Book. Sociological Methods & Research.

[55] de Reuver M, Bouwman H (2015). Dealing with self-report bias in mobile internet acceptance and usage studies. Information & Management.

