# Supplementary material for: Anxiety and Sleep Disturbances Among Health Care Workers During the COVID-19 Pandemic in India: Cross-Sectional Online Survey
Source: JMIR Public Health Surveill. 2020 Dec 22;6(4):e24206. doi: 10.2196/24206 (PMC7758087; doi:10.2196/24206)
Supplement: Multimedia Appendix 1 [file publichealth_v6i4e24206_app1.pdf]

Healthcare workers' survey

The whole world has been severely affected by Coronavirus Disease 2019(COVID-19) since December, 2019. Healthcare workers are at highest risk of developing COVID-19 infection being the frontline workers in this scenario. Risk to own families, shortage of personal protective equipment (PPE) as well as lack of clarity about pathogenesis, routes of transmission and treatment modalities for COVID-19 infection are further confounding factors adding to the stress of working in this environment. We aim to assess the mental health burden of healthcare professionals during the outbreak using a web-based cross-sectional survey.

Our survey aims to provide data support for accurately understanding the level of mental health burden amongst healthcare workers during the present COVID-19 outbreak. Our team will be grateful for your anonymous participation to enable better understanding of this burden on healthcare workers.

Over the last two weeks, how often have you been bothered by the following problems?

Please select one of the 4 choices for the symptoms given as below

* Required

1. Feeling nervous, anxious, or on edge *

   Mark only one oval.

   - Not at all
   - Several days
   - More than half the days
   - Nearly every day

2. Not being able to stop or control worrying *

   Mark only one oval.

   - Not at all
   - Several days
   - More than half the days
   - Nearly every day

3. Worrying too much about different things *

Mark only one oval.

- Not at all
- Several days
- More than half the days
- Nearly every day

4. Trouble relaxing *

Mark only one oval.

- Not at all
- Several days
- More than half the days
- Nearly every day

5. Being so restless that it is hard to sit still *

Mark only one oval.

- Not at all
- Several days
- More than half the days
- Nearly every day

6. Becoming easily annoyed or irritable *

Mark only one oval.

- Not at all
- Several days
- More than half the days
- Nearly every day

7. Feeling afraid, as if something awful might happen *

Mark only one oval.

- Not at all
- Several days
- More than half the days
- Nearly every day

8. If you checked any problems, how difficult have they made it for you to do your work, take care of things at home, or get along with other people? *

Mark only one oval.

- Not difficult at all
- Somewhat difficult
- Very difficult
- Extremely Difficult

9. Over the last 7 days, please think about the quality of your sleep overall, such as how many hours of sleep you got, how easily you fell asleep, how often you woke up during the night, how often you woke up earlier than you had to in the morning and how refreshing was your sleep. During the past 7 days, how would you rate your sleep quality overall on a scale of 0 to 10 with 0 being terrible and 10 being excellent? *

Mark only one oval.

- 1
- 2
- 3
- 4
- 5
- 6
- 7
- 8
- 9
- 10

10. Do you have access to adequate quality and quantity of Personal Protective Equipment (PPE)? *

Mark only one oval.

- Yes
- No
- Not sure
- PPE not required for my duties

11. If you don't have access to adequate quality/quantity of PPE, do you feel anxious/reluctant/apprehension to perform your duties when requiring patient contact *

Mark only one oval.

- ◯ Yes
- ◯ No
- ◯ PPE doesn't matter to me. Patient care is more important
- ◯ PPE not required for my duties

12. Your age *

Mark only one oval.

- ◯ Less than 30 years
- ◯ 30 to 44 years
- ◯ 45 to 60 years
- ◯ Above 60 years

13. Your country of residence *

____

14. Your gender *

Mark only one oval.

- ◯ Female
- ◯ Male
- ◯ Prefer not to say

15. Your profession *

Mark only one oval.

- Doctor
- Nurse
- Paramedic
- Dentist

16. Marital status *

Mark only one oval.

- Married
- Married with children
- Unmarried
- Divorced
- Prefer not to state

17. Educational level *

Mark only one oval.

- Intermediate
- Graduate
- Postgraduate
- Doctorate

18. What is the type of healthcare facility where you work? *

Mark only one oval.

- ( ) Primary
- ( ) Secondary
- ( ) Tertiary
- ( ) Not a healthcare facility
- ( ) Retired
- ( ) Other: ____________

19. Your field of specialization, if any. (Please mention if specialised in non-health related field as well OR not specialised) *

____

Google
